# Supplementary material for: Energy and cost efficiency of Bitcoin mining endeavor
Source: PLoS One. 2023 Mar 30;18(3):e0283687. doi: 10.1371/journal.pone.0283687 (PMC10062675; doi:10.1371/journal.pone.0283687)
Supplement: Attachment — Submitted filename: 2023-02-17_Response-to-Reviewers_PlosONE.pdf [file pone.0283687.s002.pdf]

Responses to Reviewers' Reports and Editor's Suggestions

Journal: PlosONE

Title: Energy and cost efficiency of bitcoin mining endeavor

We are pleased to express our gratitude for the editor's recommendation as well as the helpful and insightful comments made by the three reviewers on our earlier version of the manuscript. In this revision, we have made every effort for responding to the comments from the three reviewers. This response letter includes four parts. The first describes the main changes of the revision. The second, third, and fourth cover the comments of Reviewer 1, Reviewer 2, and Reviewer 3, respectively. We believe that the paper has improved as a result of these changes.

Note that, for difference, the original comments of the reviewers are presented as *italic words with lower fonts* in this response letter. Additionally, the corresponding responses are presented as black words. Finally, the quoted contents in this revision are presented with a yellow background.

Part I: Summary of major changes in this version

1. Since all the comments from the editor and three reviewers are very helpful and constructive, our revision has followed almost all of their comments and suggestions.
2. We have added contributions to this paper and we have indicated what is new in our approach and why such research adds value.
3. We have addressed the main policy implications of this study and we referred to the problem of generalization of our results for other cryptocurrencies.
4. We comprehensively revised the methodological section introducing supplementary explanations in order to increase its readability.
5. We have added additional rationale for the use of selected variables and clarification of the meaning of the crucial variables and metrics used in this study.
6. We added a discussion of the existing literature in order to improve the motivation of the paper.
7. We have corrected all the notes under the Tables and Figures presented in this paper.
8. We have additionally expanded the *Conclusions* section in order to outline a summary of the findings, contributions, and implications of this study.
9. We clarified the meaning of BTC translation fees (section 3.9)
10. We have corrected numbers in Figure 17 and in Table 3 because the value for '*USD profit before hardware*' was replaced by '*USD profit after hardware*'.
11. We have added the correlation matrix between the main variables affecting the results of the final profitability model.
12. The additional clarification and explanation of RQ7, RQ8, and RQ9 was added.
13. We have carefully read the whole paper and we introduced numerous corrections with regard to its content, structure, and flow of text.
14. Additionally, we sent the manuscript for professional proofreading, and once again we checked all typos, spelling errors, and grammatical mistakes. All changes are marked in the version with track changes mode. All corrections are indicated in the version with track changes mode.

Part II: Response to the comments of Reviewer 1

Reviewer 1 suggested nine comments, which we rewrote and responded them respectively, as follows.

Comment 1

The Introduction should further motivate the study. Why this study is necessary? What policy level problem this study is addressing? How the study is expected to provide any solution to that problem? How the choice of sample is complementing that problem? Are the results and policies generalizable? Introduction is silent in all these aspects. Mere choice of new variables, new methods, or choosing a new context are not considered as contribution of a study. Try to find out the policy level problem, as academic literature will not be able to provide you with the specific policy issue.

Response to Comment 1:

Thank you for these important comments. Below please find our answers to each partial question raised in Comment #1:

(a) *Why this study is necessary? ->*

We answer this question at the beginning of the *Introduction* section (1st section) in its first paragraph:

The main rationale for this paper is to address a collective misunderstanding in the matter of cryptocurrency mining efficiency which has become increasingly persistent in many sources like new mining initial coin offering (ICO) white papers, cryptocurrency news, blogs, online profitability calculators, etc. Our aim is to provide a meticulous analysis of the Bitcoin (BTC) mining efficiency when the rapid growth of mining difficulty and Bitcoin network hash rate are driving mining machines' participation rate to quickly erode in time. We restrict our analysis of cryptocurrency mining efficiency solely to the Bitcoin network leaving all the other networks for further research.

(b) *What policy level problem this study is addressing ->*

We answer this question at the beginning of the *Introduction* section (1st section) in its second paragraph:

We refer to the issue of mining activity also from the macro-policy level addressing such important topics as the efficiency of the use of various energy sources and trying to answer the question of what could be the consequence of prolonging the unprofitability of mining activity for the whole ecosystem of cryptocurrencies.

(c) *How the study is expected to provide any solution to that problem? ->*

We answer this question at the end of this paper in the Conclusion section (8th section) by referring to the main research hypothesis and then by providing the answer to detailed research questions (RQ1-RQ9) raised in this study:

The research we have presented allows us to tackle our main hypothesis and additionally to answer several research questions. With no doubt, we can refer to our main research hypothesis (RH1): Is BTC mining profitable under current mining conditions? Under current market conditions mining is not profitable but is very close to be profitable. Detailed calculations which enable us to formulate such answers are presented in previous sections. The problem is that such an answer raises many other questions connected with the overall blockchain and cryptocurrency environment which require further research. Below we will refer to all of them one by one.

(d) How the choice of sample is complementing that problem? ->

The issue was not exactly the case in our study because our analysis regarded the profitability of BTC mining at a certain point in time. Therefore, we prepared all the necessary data required for such an analysis as is described in detail in the *Crucial assumptions and data* section (section 3), and then we performed a detailed sensitivity analysis of various possible scenarios in order to make our conclusions stronger and introduce some robustness checks.

(e) Are the results and policies generalizable? ->

We answer this question at the beginning of the *Introduction* section (1st section) at the end of its first paragraph:

Nevertheless, our analysis and the methodology used can be generalized to other cryptocurrencies, which use proof-of-work and can be treated as a state-of-the-art approach to define when mining is efficient taking into account all crucial characteristics of the given cryptocurrency.

(f) *Mere choice of new variables, new methods, or choosing a new context are not considered as contribution of a study. Try to find out the policy level problem, as academic literature will not be able to provide you with the specific policy issue.*

We refer to this issue in the *Introduction* section (1st section) at the end of its second paragraph:

As far as we know, this is the first attempt to analyze the issue of BTC mining in such a thorough way. Until now, the scientific literature does not provide us with reliable and complete models, with comprehensive "what-if" scenarios of mining activity. Additionally, we used the largest possible number of external variables which can affect the adequacy of our methodology and its potential for generalization for other cryptocurrencies.…

Comment 2:

In the introduction and literature review, you need to discuss the existing literature to improve the motivation of the paper and broaden the view of readers and display the contribution of this paper. You may consider the following papers https://doi.org/10.1016/j.energy.2022.124172; https://doi.org/10.1016/j.rser.2022.113058

Response to Comment 2:

Thank you for this important comment. We addressed this problem by adding a discussion of these two papers in the *Literature review and business mining activities review* section (section 2):

There were also some other papers, indirectly referring to mining activity through analysis of energy shocks and their co-movements with carbon market dynamics (Ren et al. (2022)) or by referring to the causality between CPU and traditional energy and green markets (Ren et al. (2023)).

Comment 3:

What is the aim of the review of literature? The authors have merely listed out the studies without even creating a debate among them. Without that debate and thoughtful contradictions, the research gap cannot be substantiated.

Response to Comment 3:

Thank you for this important comment. We have added the relevant discussion at the end of the first part of the *Literature Review* section (section 2) before section 2.1.

The presented literature review shows that there were some attempts to the issue of mining activity and its profitability but they only partially referred to the main subject or were published a quite long time ago. Moreover, none of these works covered the subject of BTC mining efficiency thoroughly enough in order to refer to all our hypotheses and research questions and this was the reason why we decided to undertake this topic.

Comment 4:

Why should the authors use this method? You need to provide more comparison analysis with other methods. Why should the authors use this method? You need to provide more analysis about the economic reasoning.

Response to Comment 4

Thank you for this important comment. We tried to explain this issue more briefly in the *Crucial assumptions and data* section (section 3) and *Methodology* section (section 4). Our model is based on the logic of the BTC blockchain and is adapted to the dynamic behavior of its main constituents and parts.

Comment 5:

The tables are not clear and the authors should add the notes under each table to explain the meanings.

Response to Comment 5:

Thank you for this important comment. We have checked if all Tables and Figures have detailed Notes. If they were omitted we have added them. Currently, all Figures and Tables are associated with notes which provide details about the contents and necessary explanations.

Comment 6:

Empirical findings – please link your findings more strongly to the: (i) theory, (ii) empirics, (iii) context; and (iv) highlight their economic, academic/research and policy implications. Closely link up and cite the papers that you have discussed in the background, theory and empirical literature review & and hypotheses development section to the findings you are presenting here.

Response to Comment 6:

Thank you for this important comment. We have linked our results to the theory, and empirics, and we have highlighted their economic and policy implications. The relevant discussion of the above-mentioned issues was added in Sections 5, 6, and 7 and it is indicated in the version of the text in track changes mode.

Comment 7:

More robust analysis should be provided.

Response to Comment 7:

Thank you for this important comment. We added additional information in the *Sensitivity Analysis* section (section 6) in order to refer to the robustness of our approach and its sensitivity to various parameters' assumptions. in order to refer to these issues adequately, the *Sensitivity Analysis* section is divided into two subsections: 6.1: *One-factor analysis ceteris paribus all*, and section 6.2: *Two-factors ceteris paribus all others,* where we refer to the robustness of the proposed methodology to single parameters and to pair of crucial parameters assumed at the beginning of the study. These parameters regard crucial state coordinates of the mining activity.

Comment 8:

Conclusion – Please outline a summary of findings, contributions, implications, limitations and avenues for future research. Especially, expand the discussions relating to implications, limitations and avenues for future research.

Response to Comment 8:

Thank you for this important comment. We have additionally expanded the *Conclusions* section (section 8), once again checking if the issues raised by Reviewer are covered.

Comment 9:

Finally, this manuscript needs careful editing by someone with expertise in technical English editing paying particular attention to sentence structure so that the goals and results of the study are clear to the readers. Please there are considerable number of typos, spelling errors and grammatical mistakes throughout the paper that a careful reading will help you to eliminate them.

Response to Comment 9:

Thank you for this crucial comment. We sent the manuscript for professional proofreading, and once again we checked all typos, spelling errors, and grammatical mistakes. All changes are marked in the version with track changes mode.

Part III: Response to the comments of Reviewer 2

Reviewer 2 suggested seven comments which we rewrite and respond them respectively, as follows.

Comment 1:

Motivation and contribution: Does the paper contain new and significant information that improve or build on existing research? What is theoretical, empirical and/or practical contribution of the paper?: -> The authors need to present in a more clear way the contribution of this research, given the large body of existing literature on this topic. Several previous studies focus on this topic. ***My question: what is novel? What is the contribution of this paper?***

Response to Comment 1:

Thank you for this interesting and important comment. We have added a few sentences in the *Introduction* section (section 1) referring to the issue of novelty and our contribution to the literature. The relevant content was added in Section 1, in the second paragraph:

We refer to the issue of mining activity also from the macro-policy level addressing such important topics as the efficiency of the use of various energy sources and trying to answer the question of what could be the consequence of prolonging the unprofitability of mining activity for the whole ecosystem of cryptocurrencies. As far as we know, this is the first attempt to analyze the issue of BTC mining in such a thorough way. until now, the scientific literature does not provide us with reliable and complete models, with comprehensive "what-if" scenarios of mining activity. Additionally, we used the largest possible number of external variables which can affect the adequacy of our methodology and its potential for generalization for other cryptocurrencies.

Comment 2:

The additional insight gained from the article ultimately remains unclear and should be pointed out precisely. After the literature review, the article merely summarizes succinctly: "The point is that none of these works covered the subject of BTC mining efficiency thoroughly enough in order to refer to all our hypotheses and research questions and this was the reason why we decided to undertake this topic."

Response to Comment 2:

Thank you for this insightful and important comment. We have decided to add additional clarification to this issue at the end of the *Literature review* section (section 2.1):

The presented literature review shows that there were some attempts to the issue of mining activity and its profitability but they only partially referred to the main subject or were published a quite long time ago. Moreover, none of these works covered the subject of BTC mining efficiency thoroughly enough in order to refer to all our hypotheses and research questions and this was the reason why we decided to undertake this topic. The issues which require further investigation are as follows: detailed sensitivity analysis of the complex profitability of BTC mining, linking the characteristics describing the BTC blockchain with mining activity, and the use of various energy sources in this process.

Comment 3:

In the third chapter, a number of metrics are introduced without deeper reflection. It remains unclear how they relate to the overall model. Even more critically, descriptive data for individual metrics are reported and discussed. Thus, there is no clear separation between reflective introduction of the methodology, documentation of results and the discussion of the article's contributions. Even more surprising is the fact that the fourth chapter introduces the methodology, which further complicates the readability of the article. The structure of the article should therefore be comprehensively revised and adapted to scientific standards.

Response to Comment 3:

Thank you for this interesting comment. The metrics introduced in the *Crucial assumptions and data* section (section 3) describe the state of the BTC blockchain affecting the profitability of mining activity and

then they are used in the *Methodology* section presenting the model evaluating the profitability of BTC mining. We visualize these metrics in the third section in order to give the reader intuition about their past fluctuations. and make it possible to understand the dynamics of the BTC network. Thus, section 3 constitutes the introduction to the methodological part presented in the *Methodology* section (section 4). The division of methodology in these two parts is crucial from the point of view of potential understanding of the whole dynamics of the variables' relations described by the equations from (6) to (18). Nevertheless, we read the whole methodological part (sections 3 and 4), and we comprehensively revised it introducing supplementary explanations in order to increase its readability. The relevant insertions were added in sections 3 and 4, and are indicated in detail in the version with track changes mode.

Comment 4:

The role of numerous variables and their calculation remains unclear. This applies, for example, to transaction cost fees, which are mentioned in rudimentary form in several places but never discussed in more detail. As a result, many parts of the article seem strangely fragmented and a good reading flow is hardly possible.

Response to Comment 4:

Thank you for this interesting comment. After a detailed analysis of all places where we refer to transactional fees and other unclear descriptions of variables, we have added additional clarification of their meaning. The relevant clarification of BTC transaction fees is added in section 3.9:

BTC fee should be understood as the total value of BTC paid by the participant using the BTC network in order to perform various activities connected mostly with BTC transfers and confirmations. These fees are earned by the "miners" and they increase the profitability of the mining process. Most often we use this variable in percentage terms, dividing "BTC transaction fees per day" by "BTC mined per day".

Comment 5:

It is at least surprising that neither the energy mix (only different cost levels) nor environmental aspects of Bitcoin mining are addressed in more depth.

Response to Comment 5:

Thank you for this insightful comment. It is especially important in our study because the cost of energy places a crucial role in the profitability of mining activity. We additionally stressed this issue in the *Sensitivity Analysis* section (subsection 6.1.iv) and in the *Breakeven* section (subsection 7.2). Various sources of energy which can be used in BTC mining activity are described in the *Literature* review section (subsection 2.1).

Comment 6:

In Figure 17, the light gray bars are obviously interchanged with the dark gray bars in the legend. Otherwise, there would be a clear contradiction to the documented results.

Response to Comment 6:

Thank you for this valuable comment. It is true, we have checked it once again and corrected the colors in Figure 17 and the values in Table 3.

Comment 7:

Overall, the article is in great need of revision, both in terms of content and linguistic quality. I suggest that a clearer structure including the streamlining of the research questions will be created, that each section should is revised with regard to its contribution to the content, and that the overall precision of the explanations will be significantly increased. I would also suggest to drop some of the figures as they do not add value to the research outcome of the article. Finally, the paper should be thoroughly proofread by a native speaker to eliminate expression and grammatical weaknesses.

Response to Comment 7:

Thank you for this interesting comment. We have carefully read the whole paper and we introduced numerous corrections with regard to its content, structure, and flow of text. Additionally, we sent the manuscript for professional proofreading, and once again we checked all typos, spelling errors, and grammatical mistakes. All changes are marked in the version with track changes mode. All corrections are indicated in the version with track changes mode.

Part IV: Response to the comments of Reviewer 3

Reviewer 3 after a long description of our paper: "*The work is just a descriptive analysis article for gathered data about mining process, nothing exceptional to be published in this journal . Although the authors try to get close to the reality, the "current conditions of BTC mining (as of 2021/12/03) in the main worldwide jurisdiction" make the whole analysis just a mirror of the situation in that specific date where the base case scenario assumes a value of BTC of 53671.61 USD. This creates a distortion on the perception of the value of the BTC because of the drawdown of the last year with the current price 16,790.40 USD, so this makes this paper difficult for forecasting predictions. The authors clarify at the break even conditions the BCTUSD price where the break even value is 61312.91 USD, which in this level was reached in October 2021 and stayed for one month above that level in the overall history of bitcoin. The question is, if we produce bitcoin by mining, which is a very slow process, can we afford the cost to produce them and wait until we reach the requested level in order to sell? Another condition, at ceteris paribus with the other, is the energy cost, from the tittle of the article, it is easy to understand which are the states have a low kWh price, with the break even decided at level of 0.05 kWh, but there no comparable analysis with other models of production of the bitcoins. The states have adapted the jurisdiction in different ways, so we cannot make the same assumptions. The sensitivity analysis is done by changing one variable, at ceteris paribus or, by changing the other two which is practically fixing the level of one variable and estimating the other but there is a need to compare the results with another paper."* suggested five comments which we rewrite and respond them respectively, as follows.

Comment 1:

Study the correlation between BTCUSD , energy cost, hardware cost and the other parameters all together, to understand their impact in the PnL% by creating a model and study their elements.

Response to Comment 1:

Thank you for this important and very interesting comment. We have added the correlation matrix of all variables which have the form of time series inside the *Crucial assumptions and data* section (section 3) and we referred to this data with regard to their potential impact on BTC mining profitability. Moreover, we addressed the impact of changes in each crucial variable in the *Sensitivity* Analysis section (section 6) and in the Breakeven section (section 7)

Comment 2:

Compare the results with other authors in blockchain industry, perhaps with different cryptocurrency like Ethereum.

Response to Comment 2:

Thank you for this interesting comment. The point is that Ethereum is currently introducing a 'Proof-of-Stake" protocol instead of 'Proof-of-Work' which is currently used in the case of BTC, Therefore, such a comparison would be hardly possible.

Comment 3:

Clarify the answer to RQ7 at the conclusion.

Response to Comment 3:

Thank you for this interesting comment. We added some clarification to RQ7 at the Conclusion section (section 8) in the part devoted to RQ7:

The answer is definitely NO, unless you are a hardware mining producer thus having a synergy effect. The better investment option for a potential miner/investor is to turn toward the BTCUSD spot or derivative market and open a position strictly dependent on the increase in the price of BTC. In such a way, the potential investor can gain from a 'pure' BTC price increase while such a trade will not depend on other characteristics specific to BTC mining activity, like energy and hardware costs, BTC network hash rate, or BTC network difficulty.

Comment 4:

RQ8: The hardware cost changes from states to state, and there is a need to compare between different machines.

Response to Comment 4.

Thank you for this interesting comment. We are aware of this fact, therefore after careful investigations of possible options with regard to the BTC mining machine we have selected the most efficient one. We take into account the combination of its price, amortization period, hash rate, and energy consumption during the mining process. We added some additional clarification devoted to this issue in the section describing the characteristics of the BTC mining machine used in this study (section 3.3). The currently modified content describing this issue is as follows:

We assumed that Antminer S19J Pro produced by Bitmain (https://www.bitmain.com/) is currently the most efficient and widely used BTC mining machine, and for sure much better than its predecessor Antminer S19 used widely in 2017, 2018, and 2019. We take into account the combination of its price, amortization period, hash rate, and energy consumption {during the mining process. We would like to stress that it does not mean that there are no other mining machines (https://news.bitcoin.com/innosilicons-t2-turbo-bitcoin-miner-is-powerful-but-gmos-b3-is-still-the-champ as of date 2021/12/03) that are more efficient with regard to single characteristics describing the mining efficiency (hash rate, energy consumption or price), but we have just selected the most popular and efficient one in our view, taking all these parameters simultaneously into account. It additionally means that if we had performed an additional level of sensitivity analysis only with regard to various types of BTC mining machines then the profitability of BTV mining would be at best lower than that reported in this study.

Comment 5:

RQ9, the options market for cryptocurrency is out of the purpose of the paper.

Response to Comment 5:

Thank you for this comment. The options market was mentioned here only as an example of a derivative instrument whose price depends on the BTC price. However, taking into account the reviewer's suggestion, we have removed the options from the reference to RQ9. The relevant changes were inserted in the *Conclusion* section (section 9) in the paragraph referring to RQ9.
